# LW1497, an Inhibitor of Malate Dehydrogenase, Suppresses TGF-β1-Induced Epithelial-Mesenchymal Transition in Lung Cancer Cells by Downregulating Slug

**DOI:** 10.3390/antiox10111674

**Published:** 2021-10-24

**Authors:** Hyun Ji Kim, Mi Kyung Park, Hyun Jung Byun, Minkyoung Kim, Boram Kim, Lu Yu, Tuan Minh Nguyen, Thi Ha Nguyen, Phuong Anh Do, Eun Ji Kim, Ji Hyun Kim, Enkhmend Enkhtaivan, Kyung Sung Kim, Ji Yun Jang, Gyeoung Jin Kang, Ho Lee, Misun Won, Kyeong Lee, Jungsook Cho, Chang Hoon Lee

**Affiliations:** 1 BK21 FOUR Team and Integrated Research Institute for Drug Development, College of Pharmacy, Dongguk University, Seoul 04620, Korea; ev4444@dongguk.edu (H.J.K.); mkpark@ncc.re.kr (M.K.P.); bhj1052@dongguk.edu (H.J.B.); kyoung2k@dongguk.edu (M.K.); boram1146@dongguk.edu (B.K.); rqftgads53730@163.com (L.Y.); tuank67a5@gmail.com (T.M.N.); nguyenha221294@gmail.com (T.H.N.); phuonganh3110dkh@gmail.com (P.A.D.); kim00662@umn.edu (E.J.K.); zlzon4018@naver.com (J.H.K.); enhmend.1771@gmail.com (E.E.); star0661@naver.com (K.S.K.); yun9230@ncc.re.kr (J.Y.J.); kaylee@dongguk.edu (K.L.); neuroph@dongguk.edu (J.C.); 2 National Cancer Center, Goyang-si 10408, Korea; ho25lee@ncc.re.kr; 3 Lillehei Heart Institute, University of Minnesota, Minneapolis, MN 55455, USA; kang0268@umn.edu; 4 Personalized Genomic Medicine Research Center, KRIBB, Daejeon 34141, Korea; misun@kribb.re.kr

**Keywords:** malate dehydrogenase, epithelial-mesenchymal transition, hypoxia inducible factor-α1, slug, A549, local oxygen tension, LW1497

## Abstract

LW1497 suppresses the expression of the hypoxia-inducing factor (HIF)-1α inhibiting malate dehydrogenase. Although hypoxia and HIF-1α are known to be important in cancer, LW1497 has not been therapeutically applied to cancer yet. Thus, we investigated the effect of LW1497 on the epithelial-mesenchymal transition (EMT) of lung cancer cells. A549 and H1299 lung cancer cells were induced to undergo via TGF-β1 treatment, resulting in the downregulation of E-cadherin and upregulation of N-cadherin and Vimentin concurrently with increases in the migration and invasion capacities of the cells. These effects of TGF-β1 were suppressed upon co-treatment of the cells with LW1497. An RNA-seq analysis revealed that LW1497 induced differential expression of genes related to hypoxia, RNA splicing, angiogenesis, cell migration, and metastasis in the A549 lung cancer cell lines. We confirmed the differential expression of Slug, an EMT-related transcription factor. Results from Western blotting and RT-PCR confirmed that LW1497 inhibited the expression of EMT markers and Slug. After orthotopically transplanting A549 cancer cells into mice, LW1497 was administered to examine whether the lung cancer progression was inhibited. We observed that LW1497 reduced the area of cancer. In addition, the results from immunohistochemical analyses showed that LW1497 downregulated EMT markers and Slug. In conclusion, LW1497 suppresses cancer progression through the inhibition of EMT by downregulating Slug.

## 1. Introduction

Lung cancer is the most common cause of cancer-related deaths worldwide [1]. Approximately 85% of lung cancer cases are due to non-small cell lung cancers (NSCLC), the most common of which is adenocarcinomas [2,3]. Metastasis is the leading cause of death in cancer patients. Lung adenocarcinoma, a subtype of NSCLC, is a highly metastatic disease, with approximately 22% of patients presenting with regional lymph node metastases and 57% with distant metastases at the time of diagnosis [4]. Despite early detection and the advent of immunotherapy, the overall 5-year survival rate is still not satisfactory. Therefore, to improve the prognosis of NSCLC and devise customized treatments, further studies on the mechanisms of initiation and progression of lung cancer, predictive biomarkers, drug targets, and the development of new therapeutic agents are required.

Epithelial-mesenchymal transition (EMT) is a key step in metastasis and anticancer drug resistance. Thus, only 15–20% of NSCLC patients with specific drug-sensitive mutations benefit from available targeted therapies, such as EGFR tyrosine kinase inhibitors (TKIs, erlotinib, and gefitinib) and EML4-ALK inhibitors (crizotinib). Even in these patients, acquired resistance is a major obstacle to a sustained positive outcome [5,6,7]. EMT is also thought to be involved in the immune evasion of cancer cells [8]. An analysis of tissues of primary human lung tumors alongside the adjacent bronchial-epithelial specimens and brain metastases showed a high expression of EMT-related markers in progressive primary lung cancer, particularly squamous cell carcinoma [9]. Several studies have suggested that EMT factors, including E-cadherin, HIF-1α, Twist, and Snail are associated with a poor prognosis [10]. HIF-1α is upregulated under hypoxic conditions and stimulates the EMT of lung cancer cells [11,12]. Therefore, inhibitors of HIF-1α are highly likely to improve cancer prognoses by inhibiting EMT.

LW1497 promotes the degradation of HIF-1α by increasing local oxygen tension by inhibiting malate dehydrogenase [13]. It has been reported that LW1497 suppresses colorectal cancer by targeting the cancer metabolism, but studies on the effect of LW1497 on EMT in lung cancer have not been reported yet. Thus, this study aimed to assess whether LW1497 suppresses the EMT of lung cancer cells.

## 2. Materials and Methods

### 2.1. Materials and Plasmids

RPMI1640 medium, fetal bovine serum (FBS), penicillin/streptomycin; P/S and phosphate-buffered saline (PBS) were from Welgene Inc. (Gyeongsan, Korea). TGF-β1 was purchased from R&D Systems, Inc. (Minneapolis, MN, USA). Antibodies used were: β-actin (1:5000, sc-8432, Santa Cruz Biotechnology (SCB), Santa Cruz, CA, USA), Vimentin (1:1000, #5741, Cell Signaling Technology (CST), Berkeley, CA, USA), Slug (1:1000, #9585, CST), N-cadherin (1:1000, 620920, BD Biosciences, San Jose, CA, USA), E-cadherin (1:1000, 610181, BD). Secondary antibodies used were: anti-mouse HRP (1:5000, sc-2005, SCB), anti-rabbit-HRP (1:5000, SA002-500, GenDEPOT, Barker, TX, USA), anti-rabbit-Alexa488 (1:500, A21202, Thermo Fisher Scientific Inc., Waltham, MA, USA), and anti-mouse-Alexa594 (1:500, A21203, Thermo Fisher Scientific Inc., Waltham, MA, USA).

### 2.2. Cell Culture

A549 and H1299 human lung cancer cell lines were purchased from the American Type Culture Collection (Manassa, VA, USA). All cells were maintained in RPMI1640 medium supplemented with 10% heat-inactivated FBS and 100 U/mL penicillin, and 100 μg/mL streptomycin. To prevent mycoplasma contamination, cellmaxin (final concentration 5 μg/mL, C3314, GenDEPOT) was used. The cells were grown at 37 °C in a humidified atmosphere containing 5% CO_2_.

### 2.3. RNA Preparation and Polymerase Chain Reaction (PCR)

TRIzol^®^ RNA isolation reagents (Invitrogen, Carlsbad, CA, USA) were used to prepare the total RNA according to the manufacturer’s instructions. A cDNA synthesis was performed with a First Strand cDNA synthesis kit (Promega, Madison, WI, USA). Reverse transcription PCR was performed in the 96-Well GeneAmp^®^ PCR System 9700 (Applied Biosystems, Piscataway, NJ, USA) and AccuPower^®^ HotStart PCR PreMix (Bioneer, Daejeon, Korea) with appropriate sense, and antisense primers were used according to the previous report [14]. Primer sequences were as follows: CDH2 (NM_001308176.2, F: 5’-ATCCGGTCCGATCTGCAGCC-3′; R: 5’-GTGGCCCCCAGTCGTTCAGGTA-3′; 198 bp), VIM (NM_003380.5, F: 5’-ACCAAGACACTAT- TGGCCGCCT-3′; R: 5’-CCCTCAGGTTCAG GGAGGAAAAGT-3′; 201 bp), CDH1 (NM_004360.5, F: 5′-TGCCCAGAAAATGAAAAAGG-3′; R: 5′-GTGTATGTGG- CAATGCGTTC-3′; 200 bp), SNAIL (NM_005985.4, F: 5’-GAGGACAGTGGGAAAGGCTC -3′; R: 5’- TGGCTTCGGATGTGCATCTT-3′; 247 bp), SLUG (NM_003068.5, F: 5’-TCCTGGTCAAGAAGCATT -3′; R: 5’- GAGGAGGTGTCAGATGGA-3′; 273 bp), and GAPDH (NM_002046.7, F: 5’-CTCCTCTGACTTCAACAGCGACA-3’; R: 5’-GAGGGTCTCTCTCTTCCTCTTGT -3’; 215 bp). Reaction products were visualized following electrophoresis on 1.2% agarose gel under UV illumination after staining with SafePinky DNA gel staining solution (GenDEPOT). qRT–PCR was performed to quantify the RNA expression levels on the CFX ConnectTM Real-Time PCR Detection System using SYBR green dye (BIO-RAD, Hercules, CA, USA) as described by the manufacturer and GAPDH, as a control gene, was used to normalize the gene expression data. The following primers were used for qRT–PCR: SLUG (NM_003068.5, 5′-CTT TTT CTTGCCCTCACTGC-3′ and 5′-ACAGCAGCCAGATTCCTCAT-3′).

### 2.4. RNA Preparation and RNA-Seq

A TRIzol^®^ reagent was used to extract total RNA from A549 cells. RNA concentrations and RNA purity were measured using NanoDropTM 2000 for conducting an RNA-seq analysis. The SMARTer Stranded RNA-Seq Kit (Clontech Laboratories, Inc., Mountain View, CA, USA) was used to prepare libraries from total RNA, and the poly(A) RNA Selection Kit (LEXOGEN, Inc., Vienna, Austria) was used to isolate mRNA. The cDNA synthesis and shearing were performed using the isolated mRNAs, following the manufacturer’s instruction and the Illumina indexes 1–12 were used to perform indexing. PCR was used to carry out the enrichment step and the Agilent 2100 bioanalyzer (DNA High Sensitivity Kit (Agilent, Santa Clara, CA, USA)) was used to check libraries for evaluating the mean fragment size. Quantification was performed on a StepOne Real-Time PCR System (Life Technologies, Inc., Carlsbad, CA, USA) using the library quantification kit. To perform high-throughput sequencing as paired-end 100 sequencing, HiSeq 2500 (Illumina, Inc., San Diego, CA, USA) was used and ebiogen (ebiogen, Seoul, Korea) conducted the RNA sequence analysis.

### 2.5. RNA-Seq Data Analysis

Fast QC was used to perform a quality control of raw sequencing data [15]. FASTX Trimmer [16] and BBMap [17] were used to remove adapter and low-quality reads (<Q20). TopHat was used to map the trimmed reads as the reference genome [18]. Cufflinks were performed to estimate gene expression levels using FPKM (fragments per kb per million reads) values [19]. Quantile normalization of FPKM values was performed on EdgeR within R (R Development Core Team, 2016) [20]. ExDEGA (ebiogen, Seoul, Korea) was used for data mining and graphic visualization.

### 2.6. Western Blot

The cell lines A549 and H1299 were harvested and washed twice with PBS. Cells were lysed in a RIPA buffer containing Xpert phosphatase inhibitor cocktail solution and Xpert protease inhibitor cocktail solution (GenDEPOT, Katy, TX, USA) on ice for 10 min and centrifuged at 15,000 rpm for 15 min at 4 °C [21]. Total protein concentrations of the supernatants were determined using the Pierce BCA Protein Assay Kit (Thermo Fisher Scientific Inc., Waltham, MA, USA). The protein lysates were loaded onto an 8~10% sodium dodecyl sulfate-polyacrylamide gel (SDS-PAGE) and transferred to a polyvinylidene difluoride (PVDF) membrane. The membranes were blocked in 5% skim milk for 1 h and incubated with primary antibodies at 4 °C for overnight. After incubation of the primary antibodies, the membranes were washed with TBST, followed by the HRP-conjugated secondary antibody (1:5000) at room temperature for 1 h. The bands were detected using West-Q ECL solution (GenDEPOT, Katy, TX, USA).

### 2.7. Co-Immunoprecipitation

Co-immunoprecipitation was performed according to the previous report [22]. A549 cells were lysed in IP lysis buffer and lysate (1 mg) was incubated for 1 h with IgG and 10 μg of protein A/G magnetic beads (Thermo Fisher Scientific Inc., Waltham, MA, USA) for preclearing. The lysate was incubated with 10 μg of anti-HIF-1α (PA1-16601, Thermo Fisher Scientific Inc., Waltham, MA, USA) and anti-Slug (SC-166476, Santacruz Biotechnology, Inc., Dallas, TX, USA) antibodies or normal mouse IgGs at 4 °C overnight. Protein A/G magnetic beads were used to incubate with the lysate at RT for 1 h. The supernatants were removed carefully and the immunoprecipitates were collected in a magnetic separation rack. Pellets were washed twice with PBS and the immunoprecipitates were eluted in 40 μL of 2x SDS-PAGE reducing sample buffer and analyzed by Western blot with anti-HIF-1α or anti-Slug antibodies.

### 2.8. Confocal Microscopy

The confocal microscopic analysis was done according to the previous report [23]. A549 and H1299 cells were seeded onto coverslips and treated with TGF-β1 or LW1497 for 48 h. After washing the cells with PBS, cells were fixed with 4% paraformaldehyde (PFA) for 10 min at room temperature and permeabilized with 0.5% Triton X-100 for 10 min. Cells were blocked with 3% BSA for 30 min and incubated with primary antibodies at 4 °C for overnight. After washing twice with PBS, cells were reacted with Alexa Fluor 488 or 594-conjugated secondary antibodies at RT for 1 h. After washing twice with PBS, cells were mounted with a VECTASHIELD^®^ antifade mounting medium with DAPI (H-1200, Vectorlabs, Burlingame, CA, USA) and visualized using a confocal microscope system (K1-Fluo, Nanoscope systems, Inc., Daejeon, Korea).

### 2.9. Cell Migration Assay

A multi-well chamber (Neuroprobe Inc., Gaithersburg, MD, USA) coated with 10 μg/mL fibronectin as a chemo attractant was used to perform migration assays [24]. For the migration assay, A549 and H1299 cells were suspended in a serum-free medium at 1 × 10^6^ cells/mL. Cells (25 μL) were added to the upper well and the lower well was filled with media containing 3% FBS (30 μL). Cells migrated through the 8 μm polyhydrocarbon filter and were incubated at 37 °C for 4 h. After the incubation, cells that had not migrated through the filter were removed and cells migrating through the lower surface of the membrane were stained by Diff-Quick according to the manufacturer’s instructions. The migrating cells were subsequently counted in no less than five randomly chosen fields (400×).

### 2.10. Cell Invasion Assay

Invasion assays were conducted using Matrigel-coated (0.7 μg/mL) 24-well Transwell inserts with 8.0 mm pore size filters (Corning, Glendale, Arizona, USA) [25]. Cells (5 × 10^4^ cells) were added to the inside of Matrigel-coated inserts and RPMI1640 culture media (500 μL) containing 10% FBS were added to the lower chamber. Cells were incubated at 37 °C for 24 h and the invading cells on the lower surface of the insert were fixed with cold methanol. After fixation, the cells were stained with hematoxylin and eosin (H&E stain) and five randomly selected fields were captured by microscope (400×).

### 2.11. Luciferase Reporter Assay

The CDH1 reporter plasmid was kindly gifted by Dr. Gu Kong, Hanyang University [26]. A549 and H1299 cells were co-transfected with CDH1 and renilla vector with Lipofectamine™ 2000 transfection reagent (Invitrogen, Carlsbad, CA, USA) and incubated for 48 h. Cells were washed twice with PBS and lysed in 250 μL of Passive Lysis 5X Buffer (PLB, Promega, Madison, WI, USA). Cells were incubated for 15 min at RT with shaking and centrifuged at 14,000× *g* for 15 s at 4 °C. Supernatant (20 μL) was added to the white 96-well plate (SPL, Pocheon, Korea) and the Dual Luciferase Assay System was used to perform a luciferase reporter assay (Promega, Madison, WI, USA). Renilla activity was used to normalize a luciferase activity, expressed as relative light units and luciferase activities were measured using GloMax luminometer (Promega, Madison, WI, USA).

### 2.12. Orthotopic Mouse Model

A total of 10 six-week-old SCID/SCID male mice were randomly divided into two groups (*n* = 5 in each group). A549 (1 × 10^6^) cells in 50 μL of PBS containing Matrigel (1:1) were directly injected into the left lung parenchyma of immunodeficient female NOD/SCID mice as described elsewhere [22]. Either 10 mg/kg or 30 mg/kg LW1497 or vehicle was administered by intraperitoneal (IP) injection 5 days a week. For histology studies, mice were euthanized for eight weeks after administration. The A549 cells were suspended in a 50% Matrigel solution, and 50 μL of this solution was injected into the left lateral thorax, about 1.5 cm above the lower rib line just below the inferior border of the scapula. Four weeks after the injection, the mice were euthanized, and the lungs were removed. The lungs were fixed in 10% neutralized buffered formalin and processed for histological analysis. All animal experiments were approved and performed in accordance with the Institutional Animal Care and Use Committee (IACUC) review board of National Cancer Center which is an Association for Assessment and Accreditation of Laboratory Animal Care International (AAALAC International) accredited facility that abides by the Institute of Laboratory Animal Resources guide (NCC-17-388). Five-week-old male non-obese diabetic/severe combined immunodeficient (NOD/SCID) mice were purchased from the Korea Research Institute of Bioscience and Biotechnology (KRIBB; Ochang, Republic of Korea).

### 2.13. Statistical Analysis

All data were expressed as the mean ± standard of error measurement (S.E.M.) of at least three independent experiments performed in triplicate. *p* < 0.05 was considered statistically significant.

## 3. Results

### 3.1. LW1497 Inhibited TGF-β1-Induced EMT in A549 and H1299 Lung Cancer Cells

**Figure 1:**
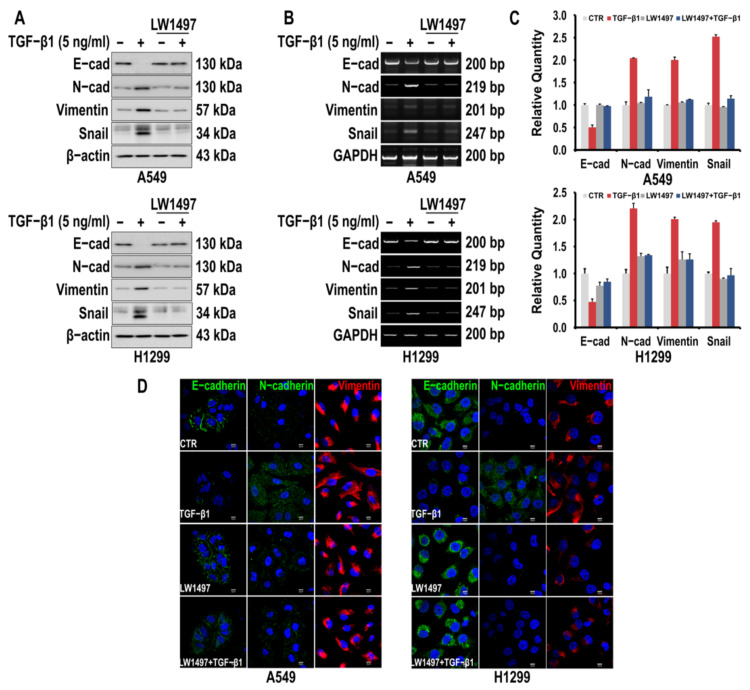
LW1497 inhibits TGF-β1-induced EMT in lung cancer cells. (**A**,**B**) A549 and H1299 cells were pretreated with 10 μM of LW1497 for 2 h and then stimulated with 5 ng/mL of TGF-β1 for 48 h. The expression levels of E-cadherin, N-cadherin, Vimentin, and Slug were measured by a Western blot (**A**) and RT-PCR (**B**). β-Actin and GAPDH levels were monitored as a loading control for whole-cell extracts. (**C**) qRT-PCR analysis of EMT markers in A549 and H1299 cells. Relative EMT marker’s expression was determined with normalization against GAPDH. To compare the ratio between the control and the experimental groups, the fold change was calculated based on the control. (**D**) Confocal microscopic analysis of EMT markers in A549 and H1299 cells. DAPI staining was used to identify the nucleus. Bar, 10 μm.

To investigate whether LW1497 inhibits the EMT of A549 and H1299 cells, the cells were first induced to undergo EMT by TGF-β1 treatment. Consequently, the epithelial marker E-cadherin was downregulated, whereas the mesenchymal markers N-cadherin and Vimentin were upregulated, as assessed via Western blotting and RT-PCR (Figure 1A,B). The TGF-β1-induced fluctuations in the levels of the EMT markers were suppressed by 10 μM LW1497 co-treatment (Figure 1A,B). Changes in the EMT markers were also confirmed by qRT-PCR (Figure 1C). The effects of LW1497 on the EMT markers were confirmed via confocal microscopy (Figure 1D).

### 3.2. LW1497 Suppressed the Stimulatory Effect of TGF-β1 on the Migration and Invasion of A549 and H1299 Cells

**Figure 2:**
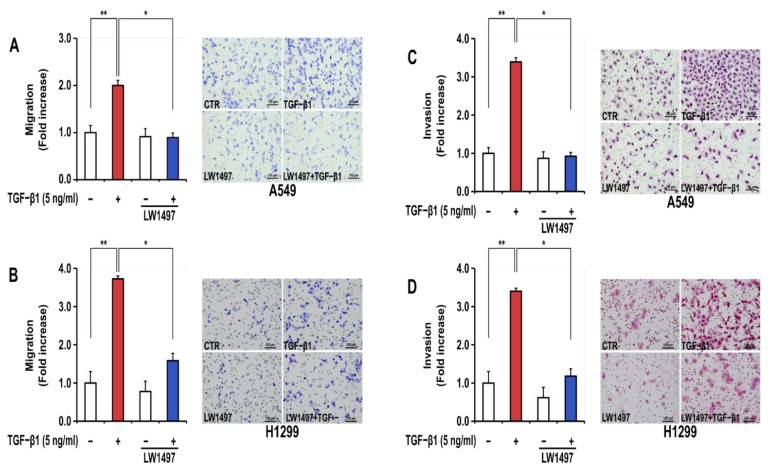
LW1497 inhibited TGF-β1-induced migration and invasion in A549 cells. (**A**,**B**) A549 (**A**) and H1299 (**B**) lung cancer cells treated as in Figure 1 were seeded on 8 μm pore Transwell chambers. Transmigrating cells were stained with Diff-Quick^®^ stain reagents and counted for each of the indicated cells. (**C**,**D**) A549 (**C**) and H1299 (**D**) lung cancer cells were added to the inside of the Transwell insert. Transinvading cells were stained with hematoxylin and eosin and counted for each of the indicated cells. Three independent experiments were performed for the results of migration and invasion assay. A * *p* value <0.05 and **, *p* value < 0.01 were considered significant and Error bars, ± SD. To compare the ratio between the control and the experimental groups, the fold change was calculated based on the control.

In general, EMT generates a phenotype of increased migration and invasion in lung cancer cells. TGF-β1 treatment significantly increased the migration and invasion of A549 and H1299 cells (Figure 2A–D). LW1497 treatment (10 μM) significantly inhibited these TGF-β1-induced increases in cellular migration (Figure 2A,B) and invasion (Figure 2C,D).

### 3.3. RNA-Seq Analysis Revealed Transcriptomic Changes Induced by LW1497 Co-Treatment in A549 Cells

**Figure 3:**
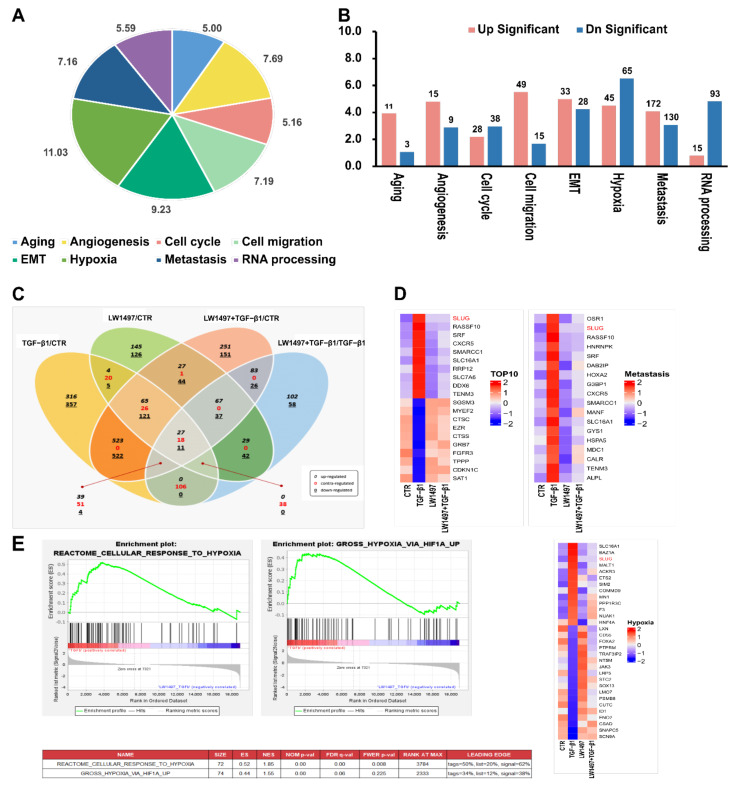
Transcriptome alteration analysis with RNA-seq in A549. (**A**) Distribution of the RNA-seq profiling annotated to the functional gene categories in LW1497 and TGF-β1-treated A549 cells. (**B**) List of enriched Gene Ontology term biological process for LW1497 and TGF-β1-treated group. Upregulated (pink) and downregulated (blue) genes were quantified. (**C**) Venn diagram of gene annotations comparisons between TGF-β1, LW1497, TGF-β1 with LW1497 and CTR. Venn diagram showing the overlap of differentially expressed genes (DEGs) specifying upregulated (black, italics), contra-regulated or genes that have diverse regulation polarity (red), and downregulated (black, underlined) genes in four pair-wise comparisons. The Venn diagram was generated using ExDEGA from E-biogen. (**D**) Heatmaps of representative top 10 up and downregulated genes, metastasis, hypoxia, and Hif1a-related genes from A549 RNA-seq results. (**E**) GSEA revealed positive enrichment of gross hypoxia via HIF1α-up and reactome cellular response to hypoxia.

To investigate how LW1497 inhibits EMT, a transcriptome analysis through RNA-seq was performed. The differentially expressed genes between A549 cells treated with TGF-β1 and those co-treated with both TGF-β1 and LW1497 are displayed in a pie graph in Figure 3A. As expected, the differentially expressed genes were enriched in genes related to hypoxia, EMT, RNA splicing, angiogenesis, cell migration, metastasis, fibrosis, and mRNA export. Of the EMT-related genes, 33 were upregulated, and 28 were downregulated (Figure 3B). Additionally, we identified the differentially expressed genes by using ExDEGA (E-biogen). Of the 738 DEGs, 294 genes were also differentially expressed in the control (CTR) group in response to the TGF-β1 treatment (TGF-β1/CTR group) (Figure 3C), suggesting that these genes are related to the effect of LW1497.

Specifically, there were 213 contra-regulated DEGs in LW1497-treated TGF-β1 compared with the TGF-β1-treated group. Of these 213 genes, 106 contra-regulated genes may be significantly associated with LW1497 treatment, because the other 107 genes were also included in LW1497/CTR and LW1497_TGF-β1/CTR groups. These 106 DEGs were regarded as candidate genes to be studied further. Their expression levels are shown in a heatmap in Supplementary Figure S1.

The top 10 up and downregulated genes were sorted according to the p-values (adjusted for multiple testing using FDR) and plotted in a heatmap, whereby the genes with the most significant expressed changes were found to be related to metastasis. Among the differentially regulated genes related to HIF-1α, the EMT-related gene Slug (SNAI2) was significantly upregulated (Figure 3D and Supplementary Table S1).

To reveal the potential action mechanisms of LW1497, we identified the pathways related to the differentially regulated genes by performing a gene set enrichment analysis. Consequently, the transcriptomic changes in the reactome-related gene set involved in the cellular response to hypoxia were identified, and the enrichment of hypoxia-related genes according to HIF-1α upregulation was observed (Figure 3E). Based on these results, we identified Slug (SNAI2) as a candidate gene associated with both hypoxia and metastasis.

### 3.4. LW1497 Inhibited TGF-β1-Induced Slug Expression in A549 Cell

**Figure 4:**
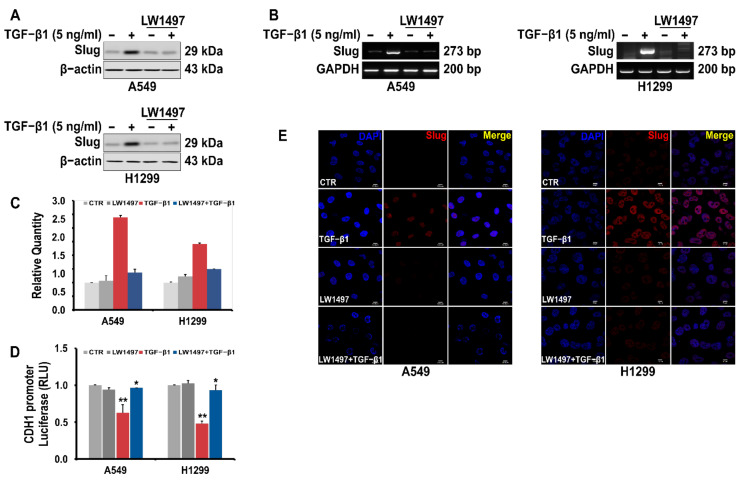
LW1497 inhibits TGF-β1-induced Slug expression in lung cancer cells. (**A**) Effect of LW1497 on TGF-β1-induced Slug expression in A549 and H1299 lung cancer cells. The expression level of Slug was measured by a Western blot, RT-PCR (**B**), and qRT-PCR (**C**). GAPDH was amplified for normalization. (**D**) Effect of LW1497 on CDH1 promoter activity. A549 and H1299 cells were pretreated with 10 μM of LW1497 for 2 h and then stimulated with 5 ng/mL of TGF-β1. After 1 day of treatment with LW1497 and TGF- β1, cells were transfected with luciferase constructs of wild-type (CDH1 pro-WT) CDH1 promoters for 24 h and the luciferase activity was measured. RLU, relative light units. * *p* < 0.05, ** *p* <0.01. To compare the ratio between the control and the experimental groups, the fold change was calculated based on the control. (**E**) Confocal microscopic analysis of the effect of LW1497 on TGF-β1-induced Slug expression in lung cancer cells. DAPI staining was used to identify the nucleus. Bar, 10 μm.

A RNA-seq analysis showed a differential expression of Slug. We confirmed this observation via RT-PCR and Western blot analyses, whereby the Slug expression in A549 cells was observed to be upregulated upon TGF-β1 treatment, and this upregulation was suppressed by co-treatment with 10 µM LW1497 (Figure 4A,B). Slug expression was also confirmed using QRT-PCR (Figure 4C). The TGF-β1-induced reduction of E-cadherin expression was recovered after treatment with LW1497 + TGF-β1 (Figure 4D). Additionally, results from the confocal microscopy showed that the Slug level in the nuclei of the cells was increased upon TGF-β1 treatment, and LW1497 co-treatment suppressed this increase (Figure 4E).

### 3.5. LW1497 Suppressed the Progression of Lung Cancer in Mice with Orthotopically Implanted A549 Cells

**Figure 5:**
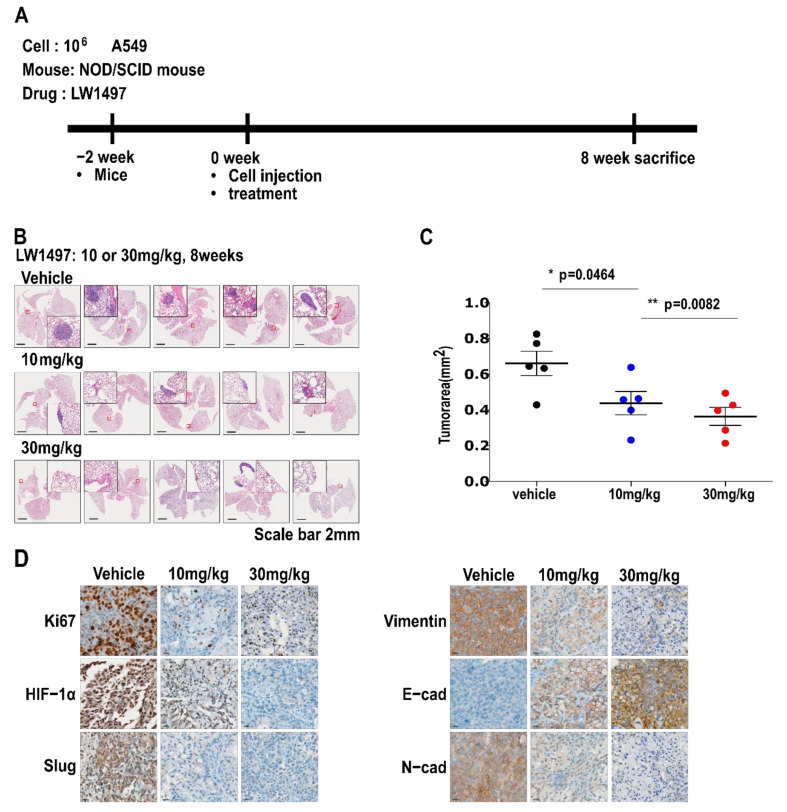
LW1497 suppressed lung cancer progression in a lung cancer orthotopic mouse model. (**A**) In vivo experimental protocol of A549 cells in NOD/SCID mouse using LW1497 drug as described in Methods section. (**B**) Hematoxylin and eosin staining of the lungs in LW1497- or vehicle-treated NOD/SCID mice injected with A549 cells. Lungs were analyzed by hematoxylin and eosin staining. (**C**) Comparison of tumor areas: mice treated with LW1497 versus mice treated with vehicle. Tumor areas were measured by intravital microscopic analysis (Axiotech Vario microscope, Zeiss, Germany). * *p* < 0.05, ** *p* < 0.01 compared with the control group. (**D**) Immunohistochemical analysis of Ki67, HIF-1a, Slug, and EMT markers including Vimentin (Vim), E-cadherin (E-cad), and N-cadherin (N-cad) in lung tumors in LW1497 (10, 30 mg/kg)- or vehicle-treated mice orthotopically injected with A549 cells into the lung.

To test whether the inhibitory effect of LW1497 on EMT can inhibit the development of lung cancer in a mouse model, we implanted 10^6^ A549 cells into the lungs of NOD/SCID mice. LW1497 was administered at 0, 10, or 30 mg/kg, and the mice were euthanized after 8 weeks. The lungs were harvested, paraffin-embedded, and then analyzed via haematoxylin and eosin (H&E) staining or immunohistochemistry (Figure 5A). Results from the H&E staining showed that the cancer area was smaller in mice treated with LW1497 (Figure 5B,C). In corroboration with our results, LW1497 treatment upregulated the epithelial marker E-cadherin and downregulated the mesenchymal markers N-cadherin, Vimentin, and Slug (Figure 5D). In addition, HIF-1α was also downregulated (Figure 5D).

## 4. Discussion

LW1497 has been reported to inhibit the expression of HIF-1α by inhibiting malate dehydrogenase. The IC_50_ value for LW1497 as an MDH inhibitor is 10 μM, so it cannot be classified as a very potent inhibitor. Therefore, more potent MDH inhibitors are needed. Since HIF-1α plays an important role in the progression of lung cancer, the effect of HIF-1α inhibitors on lung cancer was studied. Consequently, it was confirmed that EMT was inhibited through the inhibition of Slug expression in TGF-β1-induced lung adenocarcinoma cells.

During metastasis, cancer cells acquire the ability to invade surrounding tissues and subsequently spread to secondary organs [27]. The acquisition of migratory and invasive abilities by quiescent epithelial cells is associated with the acquisition of mesenchymal characteristics and concomitant loss of the epithelial phenotype, a phenomenon called epithelial-mesenchymal transition (EMT) [28]. EMT also confers resistance to anoikis and evasion from the immune surveillance and, in some cases, has been associated with the stem-cell-like properties of the resulting mesenchymal cells. All of these characteristics may be necessary for cancer cells to metastasize successfully. Therefore, the inhibition of EMT may be a reasonable strategy to prevent metastasis.

The oxygen tension (pO_2_) in physiological tissues is typically 10–80 mmHg, whereas tumors often contain regions with severe (<0.5 mmHg) or moderate (0.5–20 mmHg) hypoxia [29]. Hypoxia can have various clinical outcomes while treating cancer. Increased radiation resistance, genomic instability, angiogenesis, invasiveness, and stem-cell properties have been reported at pO_2_ levels below 1–10 mmHg [30]. Most importantly, cells may be more resilient to nutrient-poor conditions and more metastatic under hypoxia than under normoxia [29,30,31].

HIF is a major transcriptional regulator activated in response to hypoxia. It consists of the oxygen-regulated HIF-α subunit (HIF-1α or HIF-2α), which dimerizes with HIF-1β under hypoxia. HIF-1α is generally more markedly upregulated than HIF-2α. Additionally, HIF-1α upregulation occurs faster (2–24 h) and at lower oxygen levels (< 0.1% O_2_) [32]. HIF-1α is associated with various EMT transcription factors, histone modifiers [(e.g., histone lysine-specific demethylase 4B (KDM4B)], enzymes [e.g., lysyl oxidase (LOX), MMP1, and MMP3], chemokine receptors 1 and 4 (CX3CR1 and CXCR4), adhesion molecules [e.g., angiopoietin-like 4 (ANGPTL4) and L1 cell adhesion molecule (L1CAM)], and miRNA targets that promote metastasis [33].

As shown in Figure 1, TGF-β1 significantly increased the expression of Snail, which was significantly reduced by LW1497. TGF-β1 induces the Snail transcription factor and promotes HIF-1 stabilization [34,35]. Interestingly, Snail expression is promoted by hypoxic conditions and regulated via HIF-1α binding to the HRE site of the Snail gene promoter [36]. Therefore, the increase in Snail expression observed in Figure 1 can be explained by the stabilization of HIF-1 by TGF-β1. In fact, many other reports have found that Snail is a direct target for HIF-1α. In particular, Snail is a direct target for HIF-1 in the EMT of human tubular endothelial cells and hepatocellular carcinoma [37,38]. Therefore, as observed in Figure 1, LW1497 appears to suppress TGF-β1-induced EMT by reducing HIF-1α expression and suppressing the expression of Snail.

Our RNA-seq results showed that LW1497 had a significant effect on the expression of genes involved in EMT or RNA-related mechanisms affecting EMT (Figure 3). It is unclear whether these results are due to the off-target effects of LW1497 or caused by the inhibition of malate dehydrogenase and subsequent inhibition of HIF-1α expression. However, since it is well known that RNA-processing mechanisms generally adapt to hypoxia, this phenomenon may also be due to the conversion from hypoxia to normoxia by LW1497 [39].

Given that LW1497 locally increases oxygen tension [13], we hypothesized that LW1497 could inhibit EMT. As expected, LW1497 inhibited the EMT of A549 and H1299 lung cancer cells. Slug expression was highly downregulated by LW1497 (Figure 3 and Figure 4). It was also confirmed in tumor tissues (Figure 5D). Therefore, LW1497 might inhibit the metastasis of A549 cells via suppressing EMT via downregulation of Slug. However, the results of our study do not address whether metastasis occurred from one lung to another. Research is needed to examine the intravital imaging and metastasis in the lymph nodes. However, it can be expected that it will affect metastasis because fluctuations in the markers that can be considered to have occurred in the EMT required for metastasis were observed.

**Figure 6:**
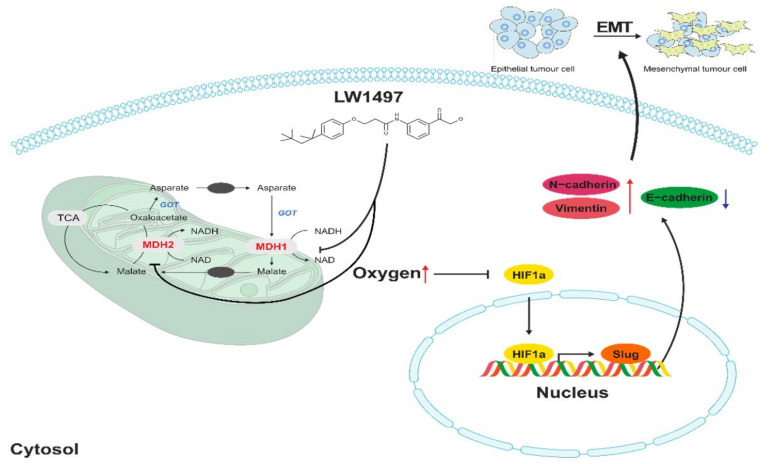
Effects of LW1497 on Slug expression. Treatment of LW1497 induces the inhibition of MDH1 and MDH2, which reduces oxygen consumption, thus increasing the concentration of oxygen locally. Thus, by inducing a decrease in the expression of HIF-1α, HIF-1 binding to HRE in the promoter site of Slug is reduced, thereby reducing the transcription of Slug. The increase in the concentration of local oxygen due to LW1497 has been reported in Naik et al. [13], while the involvement of Slug in regulating the expression of cadherins and Vimentin has been reported by Hu et al. [48].

Slug, encoded by the SNAI2 gene (previously known as Slug), is one of the three members of the Cochlear family of zinc-finger transcription factors (TFs) [40]. It is highly conserved among vertebrate species and is widely regarded as a proto-epithelial–to–mesenchymal transition TF (EMT-TF) [40,41]. As an EMT-TF, Slug promotes cell adhesion and loss of polarity while conferring the cells with migratory and invasive abilities [42]. In the case of EMT involving these Slugs, it was reported that EMT induced by low RHOB exhibits a Slug-dependent mesenchymal phenotype [43]. In addition, HIF-1α is associated with the expression of Slug in various cancers presumably because the promoter of the SNAI2 gene contains an HIF-1α response element where HIF-1α binds to and acts as a transcription factor [11,44,45,46,47]. Therefore, the LW1497-induced decrease in Slug expression is most probably because of the decreased interaction between HIF-1α and the SNAI2 gene due to the reduction in HIF-1α level caused by the increase in local oxygen tension (Figure 6).

The importance of malate dehydrogenase 1/2, the target of LW1497, in cancer has already been widely reported. For example, glutamine-dependent pancreatic ductal adenocarcinoma (PDAC) cells require MDH1 to maintain the cellular redox state by reprogramming the glutamine metabolism, and knocking down MDH1 compromises the survival of PDAC cells [49,50]. Knocking down MDH1 also inhibits fatty-acid synthesis, reducing the survival rate of BT474 cells, which are Erb-B2 receptor tyrosine kinase 2 (ERBB2)-positive breast cancer cells [51]. Upon stabilization via glucose depletion, MDH1 translocates to the nucleus and interacts with p53 to regulate the transcription of p53-dependent metabolic checkpoints [52]. In addition, overexpression of MDH2 is associated with drug resistance and short relapse-free survival in patients with prostate cancer [53,54].

In prostate cancer cells, MDH2 knockdown inhibits proliferation and enhances docetaxel sensitivity through induced metabolic inefficiency [55]. In addition, L-2-hydroxyglutarate (2-HG), an oncometabolite, is produced by malate dehydrogenase and lactate dehydrogenase under acidic conditions [56]. The 2-HG generated under acidic conditions is said to stabilize HIF-1α even under normoxia. Accordingly, malate dehydrogenase indirectly inhibits HIF-1α degradation. However, there is currently no report suggesting a direct association between MDH1/2 and EMT. However, in malic dehydrogenase, a similar enzyme, association with EMT has been reported in liver and pancreatic cancers [57,58].

## 5. Conclusions

LW1497, a malate dehydrogenase 1/2 inhibitor, blocks the TGF-**β**1-induced EMT of A549 cells via Slug downregulation and thereby suppresses the progression of the lung cancer caused by transplanted A549 cells in mice. Accordingly, LW1497 may be used as a therapeutic agent against lung cancer in the future.

## Data Availability

Data are contained within the article or Supplementary Materials.

## Supplementary Materials

The following are available online at https://www.mdpi.com/article/10.3390/antiox10111674/s1, Figure S1: Heatmap of the 106 genes that are differentially expressed between TGF-β1/CTR and LW1497_TGF-β1/TGF-β1 groups. Red: increased expression, blue: decreased expression. Table S1: The top 10 significantly down and upregulated genes, fold changes, and associated functional categories for genes from LW1497 and TGF-β1-treated A549 cells.
