# Supplementary material for: LW1497, an Inhibitor of Malate Dehydrogenase, Suppresses TGF-β1-Induced Epithelial-Mesenchymal Transition in Lung Cancer Cells by Downregulating Slug
Source: Antioxidants (Basel). 2021 Oct 24;10(11):1674. doi: 10.3390/antiox10111674 (PMC8615288; doi:10.3390/antiox10111674)
Supplement: Supplementary file 1 [file antioxidants-10-01674-s001.zip › antioxidants-1377988-Supplement table S1.pdf]

Gene	Category	Fold change		
		TGF-β1/CTR	AC1497 /CTR	AC1497+TGF-β1/CTR
CDKN1C	Aging, Cell cycle, EMT, Hypoxia, Metastasis	0.469	1.207	1.195
GRB7	Cell migration, Metastasis	0.504	0.941	1.037
MYEF2	RNA processing	0.520	1.085	1.034
SAT1	Angiogenesis, EMT, Hypoxia, Metastasis	0.423	0.919	0.819
SGSM3	Cell cycle	0.609	1.158	1.162
TPPP	Cell cycle	0.478	1.042	0.895
CTSS	Metastasis	0.505	1.036	0.905
EZR	Cell cycle, Hypoxia, Metastasis	0.506	1.011	0.895
FGFR3	Metastasis	0.478	0.802	0.831
CTSC	Aging, Metastasis	0.516	0.977	0.893
SLC16A1	Cell migration, Hypoxia, Metastasis	1.627	1.018	1.112
TENM3	Metastasis	1.508	0.880	1.027
SLC7A6	Cell cycle, Cell migration, Hypoxia	1.554	0.921	1.055
CXCR5	Cell migration, Metastasis	1.662	0.961	1.126
DDX6	RNA processing	1.551	0.895	1.027
SRF	Aging, Angiogenesis, Cell migration, Hypoxia, Metastasis	1.821	1.024	1.175
SLUG	Cell migration, EMT, Hypoxia, Metastasis	2.045	1.242	1.245
SMARCC1	Hypoxia, Metastasis, RNA processing	1.643	0.863	0.987
RASSF10	Metastasis	1.857	1.067	1.094
RRP12	RNA processing	1.559	0.826	0.907

AC1497+TGF-β1/TGFβ1
2.548
2.059
1.986
1.938
1.907
1.873
1.792
1.768
1.739
1.730
0.684
0.681
0.679
0.678
0.662
0.645
0.609
0.601
0.589
0.582
