# Supplementary figures and images for: LW1497, an Inhibitor of Malate Dehydrogenase, Suppresses TGF-β1-Induced Epithelial-Mesenchymal Transition in Lung Cancer Cells by Downregulating Slug
Source: Antioxidants (Basel). 2021 Oct 24;10(11):1674. doi: 10.3390/antiox10111674 (PMC8615288; doi:10.3390/antiox10111674)

Supplement Figure S1

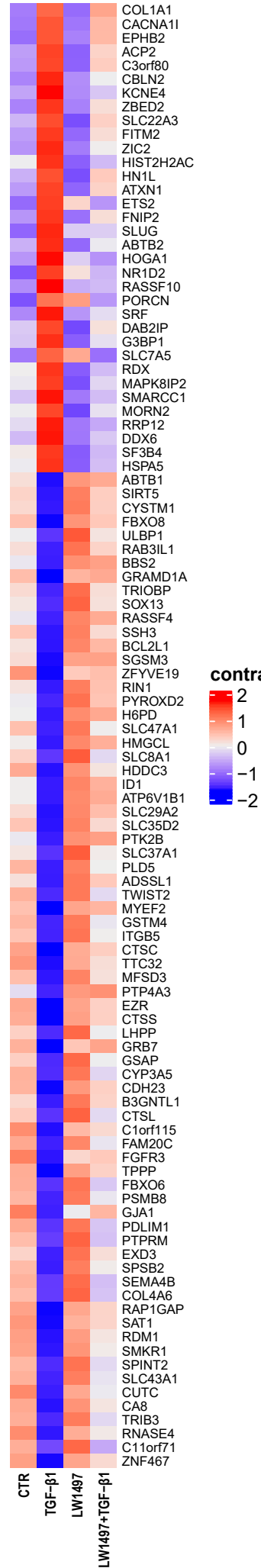

Supplement: Supplementary file 1 [file antioxidants-10-01674-s001.zip › antioxidants-1377988-supplementary figure S1.pdf]
